# intePareto: an R package for integrative analyses of RNA-Seq and ChIP-Seq data

**DOI:** 10.1186/s12864-020-07205-6

**Published:** 2020-12-29

**Authors:** Yingying Cao, Simo Kitanovski, Daniel Hoffmann

**Affiliations:** Bioinformatics and Computational Biophysics, Faculty of Biology and Center for Medical Biotechnology (ZMB), University of Duisburg-Essen, Universitätsstr.2, Essen, 45141 Germany

**Keywords:** RNA-Seq, ChIP-Seq, Integrative analysis

## Abstract

**Background:**

RNA-Seq, the high-throughput sequencing (HT-Seq) of mRNAs, has become an essential tool for characterizing gene expression differences between different cell types and conditions. Gene expression is regulated by several mechanisms, including epigenetically by post-translational histone modifications which can be assessed by ChIP-Seq (Chromatin Immuno-Precipitation Sequencing). As more and more biological samples are analyzed by the combination of ChIP-Seq and RNA-Seq, the integrated analysis of the corresponding data sets becomes, theoretically, a unique option to study gene regulation. However, technically such analyses are still in their infancy.

**Results:**

Here we introduce *intePareto*, a computational tool for the integrative analysis of RNA-Seq and ChIP-Seq data. With *intePareto* we match RNA-Seq and ChIP-Seq data at the level of genes, perform differential expression analysis between biological conditions, and prioritize genes with consistent changes in RNA-Seq and ChIP-Seq data using Pareto optimization.

**Conclusion:**

*intePareto* facilitates comprehensive understanding of high dimensional transcriptomic and epigenomic data. Its superiority to a naive differential gene expression analysis with RNA-Seq and available integrative approach is demonstrated by analyzing a public dataset.

**Supplementary Information:**

The online version contains supplementary material available at (doi:10.1186/s12864-020-07205-6).

## Background

With increasing accessibility and application of high-throughput sequencing (HT-Seq), it has become possible, in principle, to combine and integrate complex transcriptomic (RNA-Seq, [1]) and epigenomic data as a multi-omics approach to understand mechanisms of gene regulation [2]. One of the most important epigenetic regulators of gene expression are histone modifications [3]. Several types of histone modifications can change the state of the chromatin in different ways and increase or decrease gene expression.

There are many interesting applications of integrative analysis of RNA-Seq and ChIP-Seq data. For instance, the consistent co-occurrence of histone modification patterns and up- or down-regulated gene expression can improve our understanding of the “histone code” [4]; or, the comparison of histone modification states with quantitative gene expression can lead to the discovery of new enhancer regions [5]; or, expression and simultaneous occurrence of different modifications at a gene can reveal gene regulation dynamics along a developmental trajectory [6]. Separate analyses of RNA-Seq or ChIP-Seq data alone can not fully explain the complex mechanisms underlying the regulation of gene expression. Efforts to quantitatively integrate available RNA-Seq and ChIP-Seq data of histone modifications in various conditions are crucial for improving our understanding of the role of epigenetics in gene regulation.

Several computational methods have been proposed to use histone modifications for predicting gene expression [7, 8]. However, these methods generally focus on the prediction of gene expression with ChIP-Seq data of different histone modifications in one cell type or state. An important task for quantitative integration of RNA-Seq and ChIP-Seq data is the identification of genes of important biological function that are differentially expressed and therefore define cell types or states. Integration could answer questions like these: For which genes do we see *consistent* changes in expression and in histone modifications as we compare different cell types or conditions? Which genes show increased expression in combination with acquisition of activating histone modifications, or decreased expression in combination with more suppressive histone modifications?

Such genes with consistent transcriptomic and epigenomic changes are more likely to point to essential functional differences and to play an important role in cell differentiation or the development of disease.

Although identification of such genes is obviously highly attractive, and matched data sets of RNA-Seq and ChIP-Seq are increasingly available, promising technical implementations are still rare and not readily available [9]. One reason may be the sheer complexity of the data, consider e.g. that there are numerous histone marks with similar but probably not identical function, such as activating marks H3K4me3, H3K4me1, H3K36me3, H3K27ac, or repressive marks H3K9me3 and H3K27me3.

There are a few methods developed to detect genes with congruent changes in RNA-Seq and ChIP-Seq between two experimental conditions. For example, Klein et al., 2014 [10] and Schäfer et al., 2017 [11] developed approaches based on Bayesian inference of mixture models [10] and hierarchical models and clustering [11]. These early methods are a great step forward towards integrative analysis, but they still suffer from limitations, e.g. with respect to the number of genomic variables that may be analyzed, or because of the danger of losing important information in the aggregating of data. Further more, their integration [11] is based on transcript level, from a biological perspective, data integration on gene level is easier to interpret than at the transcript level.

Here we present a quantitative method for the integrative analysis of RNA-Seq and ChIP-Seq data for several different histone modifications. We frame integrative analysis as multi-objective optimization problem that we solve by Pareto optimization [12]. Multi-objective optimization has significant advantages compared to single-objective optimization, e.g., in classification, system optimization, and inverse problems [13]. With our new R package *intePareto* we provide a first solution of Pareto optimization to the integration of RNA- and ChIP-Seq data sets. Specifically, *intePareto* is a flexible and user-friendly tool (1) to match these data sets on gene level, (2) to integrate them in a quantitative fashion, (3) to examine abundance correlations of histone modifications and gene expression, and (4) to prioritize genes based on the consistence of changes between conditions in both RNA-Seq and ChIP-Seq using Pareto optimization. The result of the last step is an informative rank-ordered gene list.

We demonstrate that integration of RNA-Seq data and ChIP-Seq data by Pareto Optimization outperforms a clustering method based on Bayesian inference of a hierarchical model [11], and the analysis of RNA-Seq alone.

## Implementation

**Fig. 1:**
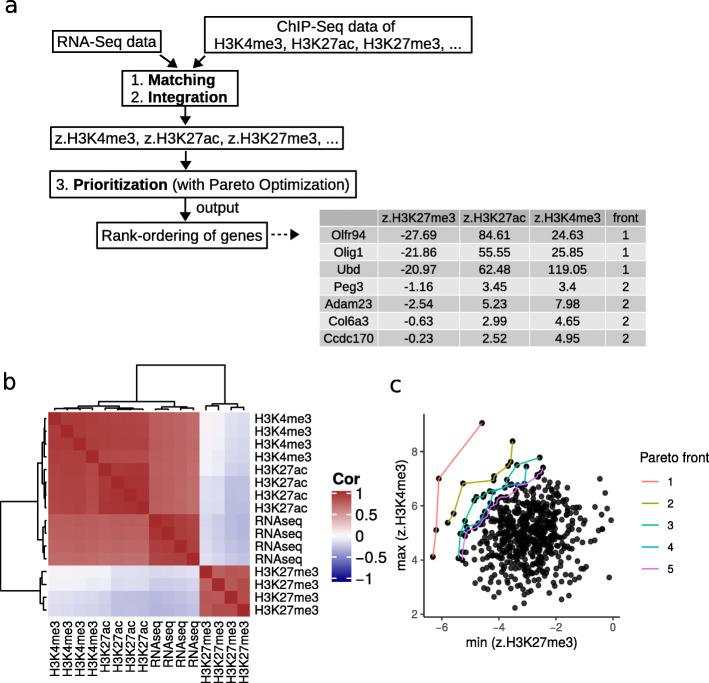
intePareto workflow. **a** The general pipeline of intePareto. **b** Heatmap of correlation matrix of RNA-Seq and ChIP-Seq for different histone marks after genewise matching. The color represents Spearman’s rank correlation across all samples. **c** Top Pareto fronts

*intePareto* is implemented as an R package that provides an easy-to-use workflow to quantitatively integrate RNA-Seq and ChIP-Seq data of one or more different histone modifications. A typical application, as presented here with 4 RNA-Seq samples and 28 ChIP-Seq samples (case study in Additional file 1), runs in less than one hour on a standard personal computer. In this section, we describe the implementation of *intePareto* in detail. The pipeline takes as first input RNA-Seq data, preprocessed by RNA-Seq quantification software, for instance estimated read counts from Kallisto [14], or other suitable quantities [15–17]. Kallisto performs well in terms of speed and quantification, so we use as input file format the output format of Kallisto. Other quantification inputs [15–17] are also accepted if structured in the same input file format. Second, the pipeline takes ChIP-Seq reads, aligned to the reference genome with tools like BWA [18], and then processed further with Samtools [19]. The workflow then comprises three main steps, 1. “Matching”, 2. “Integration”, 3. “Prioritization” sections (Fig. 1a).

### Matching

Our first problem is to link histone modification data with the corresponding gene expression data. Hence, the first step is to match quantitative histone modification data from ChIP-Seq to the biologically corresponding gene expression data as measured by RNA-Seq, or in other words: to find the target genes for histone modifications.

This matching of RNA-Seq and ChIP-Seq data is complicated by the fact that one gene usually has multiple transcripts, and multiple transcript starting sites (TSSs), which means that there are multiple promoters that can drive gene expression [20]. Another more challenging task is that the link between enhancers and genes is much more difficult to determine. Contrary to promoters that reside approximately 3 kilobases (kb) upstream from the transcription start site (TSS) of a gene, enhancers are often found dozens of kb away from the genes they influence. Moreover, enhancers are tissue- and cell type-specific and highly variable [21–23].

Several methods for predicting target genes for histone modifications have been published [24–26]. However, the lack of agreement between them discouraged us to include them in our pipeline [27].

For ChIP-Seq data of histone modification marks that are enriched in promoter regions, like H3K4me3 and H3K27me3, *intePareto* offers two matching strategies: (1) *highest* – choose the promoter with maximum ChIP-Seq abundance value among all the promoters as a representative of the ChIP-Seq signal for this gene; (2) *weighted.mean* – calculate the abundance weighted mean of all the promoters to represent the ChIP-Seq signal for this gene. In this study the promoter region was defined as 5 kb stretch with the TSS at the center; we found that this value safely included all relevant ChIP-Seq signals. This definition can be adapted if necessary.

More matching strategies will be offered in future versions with increasing availability of validated annotated enhancers and of studies that examine the relationship between the density of ChIP-Seq and expression level of RNA-Seq. After the genewise match of RNA-Seq and ChIP-Seq data, the correlation of RNA-Seq and ChIP-Seq can be examined for each histone mark (Fig. 1b)

### Integration

After the genewise matching of RNA-Seq and ChIP-Seq, these two data types are integrated by calculation of *log* fold changes (FC) between conditions, as implemented in DESeq2 [28]. For that purpose we propose to use DESeq2 because it works well for both RNA-Seq and ChIP-Seq data [29]. Another benefit is that *apeglm* algorithm [30] is used to shrink the logFC values to zero when the counts are low, dispersion is high, or the number of biological replicates is small. To normalize the data for sequencing depth and RNA composition, the median of ratios method is implemented [28]. *intePareto* determines the Z scores for each gene *g* and each histone modification type *h*, defined as:

$$Z_{g,h} = \frac{logFC^{(\text{RNA})}_{g}}{\text{sd}\left(logFC^{(\text{RNA})}_{g}\right)}\cdot \frac{logFC^{(\text{ChIP})}_{g,h}}{\text{sd}\left(logFC^{(\text{ChIP})}_{g,h}\right)}. $$

A combination of gene and histone mark has a high, positive Z score if between the compared conditions or cell populations gene expression and histone modification change strongly and in the same direction, i.e. both up or both down.

### Prioritization

*intePareto* takes the Z scores for different, user-selected histone modifications as input, so that for each gene we have several Z scores.

To this end, we can collect all Z scores in an objective function, namely the vector of the *n* Z scores (one for each histone modification), i.e. (*α*_1_*Z*_1_,*α*_2_*Z*_2_,…,*α*_*n*_*Z*_*n*_), where *α*_*i*_∈{−1,1}, depending on whether the histone mark is repressive or activating.

We can then interpret the identification of genes that show strong and consistent changes across histone marks as a multi-objective optimization problem, and we solve this problem by a Pareto optimization algorithm [12, 31].

The result is a ranking of genes in Pareto fronts. Using marks H3K27me3 and H3K4me3 as an example, genes in the first Pareto front could minimize Z scores for the repressive mark H3K27me3, and simultaneously maximize the Z scores for the activating mark H3K4me3. This simultaneous optimization is understood in the sense that genes in the first Pareto front are not *dominated* by other genes, i.e. no genes outside the first Pareto front have a lower H3K27me3 Z score and simultaneously a higher H3K4me3 Z score. The second Pareto front is determined in the same way after removal of the first Pareto front, etc. Fig. 1c shows an example of the resulting rank ordering.The Additional file 1 gives more details and an example application of *intePareto*.

## Results

### Evaluation of *intePareto* using publicly available data

#### RNA-Seq and ChIP-Seq data

We evaluate *intePareto* based on publicly available RNA-Seq and ChIP-Seq data from a study of Tet methylcytosine dioxygenase 2 (Tet2) knockout mouse embryonic stem cells (mESCs) that are compared to wild type mESCs [32]. With Tet2 assumed to be involved in the regulation of DNA methylation at enhancers, we expected to find congruent changes between the epigenomes and transcriptomes of Tet2 knockout and wildtype mESCs. For each cell type, the data consists of biologically replicated RNA-Seq data and ChIP-Seq data for histone marks H3K4me1, H3K4me3, H3K9me3, H3K27ac, H3K27me3 and H3K36me3 (see Additional file 1 for details).

#### Data processing

The raw RNA-Seq data in FASTQ format was aligned and quantified with Kallisto (version 0.43.1) [14] against a reference transcriptome downloaded from the ENSEMBL database [33]. The outputs of this step are estimated counts of reads and TPM values for each gene of a given cell condition. The raw ChIP-Seq data in FASTQ format was aligned with BWA (0.7.17) [18] also against a reference genome from ENSEMBL. The resulting files were sorted and the corresponding index files were built with Samtools (version 0.1.19) [19].

#### Analysis with intePareto

We know that the histone marks H3K4me3, H3K27me3, and H3K9me3 are enriched at gene promoter regions [34, 35]. Other marks such as H3K4me1 and H3K27ac are often associated with gene enhancers as well as active promoter regions, while H3K36me3 is associated with the gene body [34–36]. To define the epigenetic signal for marks that are prevalent at gene promoters we counted the number of ChIP-Seq reads falling into the promoter region of specific genes. For H3K36me3 we counted the total number of reads that fall into the genomic body.

**Fig. 2:**
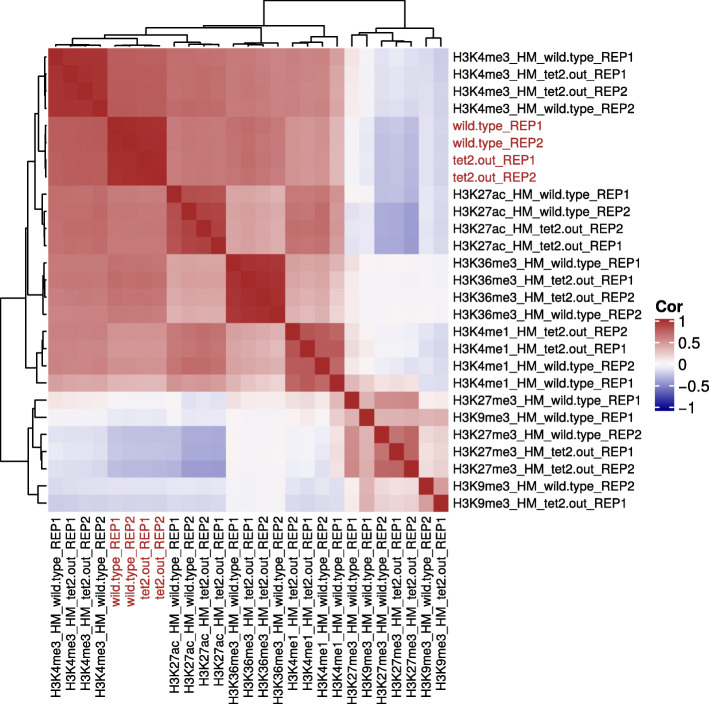
Heatmap of correlation matrix. Heatmap shows correlation matrix of all RNA-Seq and ChIP-Seq samples. Sample names marked in red are RNA-Seq samples. Sample names marked in black are ChIP-Seq samples

Matching of RNA-Seq and ChIP-Seq data was performed with *highest* strategy as described in “Implementation” section (also see Additional file 1). We demonstrate that our matching strategy captures meaningful epigenetic and transcriptomic signals, by showing that the gene expression is positively correlated with the signal of active marks, and negatively correlated with the signal of repressive marks (Fig. 2) [37, 38]. The matched data was integrated (*doIntegration* function), followed by a prioritization (*doPareto* function) based on Pareto optimization. The optimization task was devised such that it prioritizes genes having high positive Z-scores for active histone marks (H3K4me1, H3K4me3, H3K27ac, H3K36me3) and low negative Z-scores for repressive histone marks (H3K9me3, H3K27me3). The resulting list of genes were sorted according to ascending fronts (Additional file 2).

### Downstream analysis of the output of *intePareto*

**Fig. 3:**
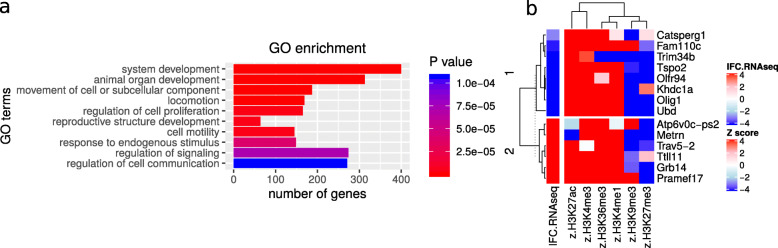
Top genes detected by *intePareto*. **a** Top 10 GO terms that are enriched for genes detected by *intePareto* in the first 5 Pareto fronts. **b** Genes in the first Pareto front. The logFoldChange of RNA-Seq (lFC.RNA-Seq) is calculated by DESeq2 in tet2 knockout condition over wild type. The Z score is calculated by *intePareto*

Gene Ontology (GO) enrichment analysis [39] of the top genes resulting from Pareto optimization by *intePareto* shows (Fig. 3a) that all enriched GO terms are known functional characteristics of Tet2 according to the data source [32] and other research. Specifically, Tet2 can influence the cell differentiation and proliferation of ESCs through altering of the methylation status of DNA, especially in neurogenic differentiation [32, 40], and the development of the heart [41, 42] and other organs [43]. Figure 3b is the heatmap of the 14 genes in the first Pareto front. There are distinct patterns between the up-regulated and down-regulated genes. The clustering dendrogram at the top of the heatmap hints at the functional similarity of H3K27me3 and H3K9me3, and the functional similarity of H3K4me1, H3K4me3, H3K27ac, and H3K36me3. This is in line with previous reports about the function of these histone marks [37, 38]. It is worth noting that the gene Eif2s3y, which was recently confirmed as strongly down-regulated [44] in Tet2 knockdown mESC, was not significantly down-regulated in RNA-Seq data alone. However, it popped up in the top two Pareto fronts of our integrative analysis. This also highlights the benefits of integrative analysis of both data types, which can reduce false negatives or false positives from analyses based on a single sample or data type.

### Comparison with existing approach

To evaluate the performance of *intePareto*, we compared our results to those of an integrative analysis with a recently published hierarchical Bayesian model-based clustering approach (“model-based approach”) [11], and to the analysis of RNA-Seq alone (Additional file 3). As quality metric for the comparisons, we used the enrichment score of interesting GO terms. For a set of genes (*G*; e.g. high-priority genes assigned to Pareto front 1), we define the enrichment score for GO term *i* as the fraction *f*_*i*_=|*G*∩*GO*_*i*_|/|*G*|, with *GO*_*i*_ the set of all genes annotated with GO term *i*.

**Fig. 4:**
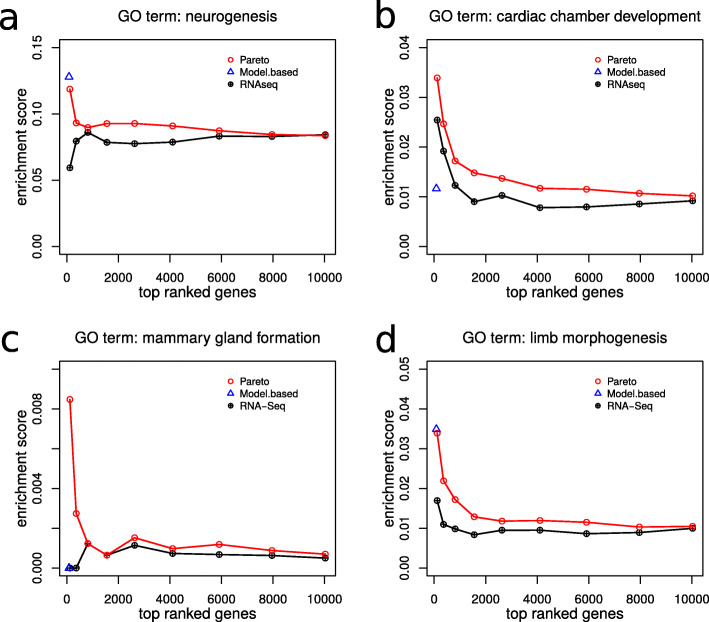
Comparison of *intePareto* with a model-based clustering approach and analysis of RNA-Seq alone. (a-d) In each of the four panels, the first point from the left on the red line marks the number of genes (x-axis) in the first two (Since there are only 14 genes in the first Pareto front shown in Fig. 3b) Pareto fronts together with the enrichment score (y-axis) of the respective GO term in that Pareto front. Accordingly, the second point refers to the genes in the first three Pareto fronts, etc. Assume that the first *i* Pareto fronts comprise a total *n*_*i*_ genes, then the corresponding point on the black line takes the first *n*_*i*_ genes, ranked by q-value obtained from the differential gene expression analysis based on RNA-Seq data alone. Note that the red line from the intePareto analysis always lies above the black line, indicating a stronger enrichment of the relevant GO terms in the integrated data compared to RNA-Seq data alone. The blue triangles mark the corresponding values of the existing integrative analysis method

The GO terms of interest were those confirmed in previous research such as “neurogenesis” [32, 40], “cardiac chamber development” [41, 42], “mammary gland formation” [43, 45], and “limb morphogenesis” [46]. Both our integrative approach and the model-based approach found that the genes in the top-ranked genes were enriched in “neurogenesis” (Fig. 4a) and “limb morphogenesis” (Fig. 4d). Analysis based on RNA-Seq alone did not find this enrichment. *intePareto* also found that the top-ranked genes are more enriched in “cardiac chamber development” (Fig. 4b) and “mammary gland formation” (Fig. 4c) as they should be. These functions were not identified by RNA-Seq analysis alone or the model-based approach. An alternative to GO enrichment, that yields complementary information, is pathway enrichment.

## Discussion and conclusions

Integrative methods such as those implemented in *intePareto* can collect more evidence from the increasing amount of HT-Seq data of different modalities, such as RNA-Seq and ChIP-Seq data. This will hopefully allow deeper insight into molecular mechanisms underlying processes like cell differentiation or disease progression. The approach chosen here can be generalized to further HT-Seq data types, e.g. from DNA methylation or chromatin accessibility.

Another use of *intePareto* lies in quality control. Specifically, the correlation matrix (Fig. 2) that is generated in the analysis procedure can be used to check ChIP-Seq data quality, which is still not straightforward [47–49]. Such quality checks prior to detailed data analysis and interpretation can avoid errors caused by low-quality ChIP-seq data, and point to possible reasons of failure.

As mentioned above, our approach can be extended in several directions. For instance, improvements are possible if the relationship between distal (even transchromosmal) regulatory elements like enhancers, and their target genes are clarified.

However, it is also true that our approach has inherent limitations. Gene regulation is of such a complexity [50–52] that it probably cannot be completely mapped on a simple approach as that proposed here. We would have to jointly consider the multitude of effects of chromatin remodelers [53, 54], transcription factor co-occupancy [55, 56], different combination of histone modification marks [4, 57], DNA methylation [58], and even RNA modifications [59, 60], which are laborious to capture and profile simultaneously [61]. Nevertheless, we think that a robust, easy-to-use approach such as *intePareto* that exploits subsets of these genomic modalities is a valuable addition to the toolbox of basic and applied genomics.

## Availability and requirements

**Project name:** intePareto**Project home package:**https://cran.r-project.org/web/packages/inteParetohttps://github.com/yingstat/intePareto (development version)**Operation system(s):** Platform independent**Programming language:** R (≥3.6.0)**License:** GPL (≥2)**Restrictions to use by non-academics:** None

## Supplementary Information

**Additional file 1** CaseStudy. Codes and details of an example application with *intePareto*. The data used in the case study are the public data we analyzed in this paper.

**Additional file 2** Results_of_intePareto. Full list of the results of integrative analysis using *intePareto*.

**Additional file 3** Results_of_RNASeq_data_analysis. Full list of the results of differential gene analysis with RNA-Seq data.

## Data Availability

All original data are available from NCBI Gene Expression Omnibus (GEO) under accession number GSE48519.

## Abbreviations

HT-Seq: High-throughput sequencing
RNA-Seq: RNA-sequencing
ChIP-Seq: Chromatin immuno-precipitation sequencing
TSS: Transcript starting sites
kb: Kilobases
TPM: Transcripts per million
Tet2: Tet methylcytosine dioxygenase 2
mESC: Mouse embryonic stem cell
ESC: Embryonic stem cell
GO: Gene ontology
