# Supplementary material for: intePareto: an R package for integrative analyses of RNA-Seq and ChIP-Seq data
Source: BMC Genomics. 2020 Dec 29;21(Suppl 11):802. doi: 10.1186/s12864-020-07205-6 (PMC7771091; doi:10.1186/s12864-020-07205-6)
Supplement: Supplementary file 1 — Additional file 1 CaseStudy. Codes and details of an example application with intePareto. The data used in the case study are the public data we analyzed in this paper. [file 12864_2020_7205_MOESM1_ESM.pdf]

Supplementary for intePareto: An R package for integrative analysis of RNA-Seq and ChIP-Seq data

Yingying Cao, Simo Kitanovski, Daniel Hoffmann

A frequent question in biology is: How do the functions of different cell types differ? E.g. we may be interested in what the effect of a mutation or gene knockout is in terms of functional differences between wild type and mutant/knockout, or how cellular function changes between two developmental stages of a cell type. One way of understanding such functional difference is to characterize them at the level of differences in repertoires in active genes or suppressed genes. The characterization of differential gene expression is helpful in this respect, but even more expressive is the combination of evidence from different experiments, namely measurements of gene expression (RNA-Seq) and measurements of various histone modifications (ChIP-Seq) that allow assessment of activation or suppression state of genes. In our experience, this combination of information gives a clearer picture of the cellular function at the molecular level than using any of the information types alone.

We have therefore developed the R package intePareto that allows such a combination of different types of sequencing data. The intePareto workflow starts with RNA-Seq and ChIP-Seq data for two different cell types or conditions. The ChIP-Seq data will in general comprise information on several histone modifications with activating or repressing function. The end product of intePareto is a list of genes prioritized according to congruence of changes of gene expression and histone modifications.

In the following we demonstrate the technical workflow with the published dataset GSE48519 where a Tet2 knockout cell line is compared to the wild type [4]. The raw data were downloaded from Gene Expression Omnibus. This set contains 4 RNA-Seq samples, 31 ChIP-Seq samples with histone modification mark of H3K4me1, H3K4me3, H3K9me3, H3K27ac, H3K27me3, H3K36me3 and control for Tet2 knockout and wild type mouse embryonic stem cells (mESCs) separately.

Preprocessing of RNA-Seq data and ChIP-Seq data

The quality of the fastq data was examined with FastQC [1]. The RNA-Seq data was then preprocessed by Kallisto [2], and ChIP-Seq data was preprocessed by BWA [5], Samtools [6]. The meta data below gives an overview of the preprocessed files that are the input files for intePareto.

```
library(intePareto)
```

```
rna_meta
```

```
##         SRR condition                                            files
## 1 SRR925874 wild.type ./data/tet2/kallisto/outputSRR925874/abundance.tsv
## 2 SRR925875 wild.type ./data/tet2/kallisto/outputSRR925875/abundance.tsv
## 5 SRR925878  tet2.out ./data/tet2/kallisto/outputSRR925878/abundance.tsv
## 6 SRR925879  tet2.out ./data/tet2/kallisto/outputSRR925879/abundance.tsv
```

```
chip_meta[1:2,]
```

```
##         SRR    mark condition                                  files
## 1 SRR925639 H3K4me1 wild.type ./data/tet2/output/SRR925639_sorted.bam
## 2 SRR925640 H3K4me3 wild.type ./data/tet2/output/SRR925640_sorted.bam
```

1. match: Match RNA-Seq and ChIP-Seq data on the gene level

Take the meta data of the preprocessed RNA-Seq and ChIP-Seq data as input. The first step of intePareto is to match the RNA-Seq data and ChIP-Seq data on the gene level. There are two strategies available now to do the matching step: (1) highest - choose the maximum promoter abundance value among all the promoters as a representative of the ChIP-Seq signal for this gene. (2) weighted.mean - calculate the weighted mean of the abundance value of all the promoters to represent the ChIP-Seq signal for this gene. Here we choose "highest":

```
library(intePareto)
chip_meta_noH3K36me3 <- chip_meta[!chip_meta$mark%in%"H3K36me3",]
res.1 <- doMatch(rnaMeta = rna_meta, # metadata of RNA-Seq
                 chipMeta = chip_meta_noH3K36me3, # metadata or ChIP-Seq
                 region = "promoter", # specify the region
                 method = "highest", # specify the strategy to do the match
                 ensemblDataset = "mmusculus_gene_ensembl"
                 # specify the dataset of corresponing species
)
chip_meta_H3K36me3 <- chip_meta[chip_meta$mark%in%"H3K36me3",]

res.2 <- doMatch(rnaMeta = rna_meta, # metadata of RNA-Seq
                 chipMeta = chip_meta_H3K36me3, # metadata or ChIP-Seq
                 method = "highest", # we don't need this parameter if we choose
                 # genebody, but it doesn't matter if we choose
                 region = "genebody", # specify the region
                 ensemblDataset = "mmusculus_gene_ensembl"
                 # specify the dataset of corresponing species
)
res.1$matched.data <- merge(res.1$matched.data,
                            res.2$matched.data)
res.1$res.chip <- merge(res.1$res.chip,
                        res.2$res.chip)
res <- res.1
```

Figure 1 shows the correlation matrix, the color represents the value of correlation coefficients of Spearman's rank correlation of all samples. From this figure we can see that RNA-Seq (wild.type__REP1, wild.type__REP2, tet2.out__REP1, tet2.out__REP2) positively correlate with active histone modification markers (H3K4me3, H3K27ac, H3K4me1, and H3K36me3), and negatively correlate with repressive histone modification markers (H3K27me3 and H3K9me3). This can confirm our match strategy works well for the match of RNA-Seq and ChIP-Seq data on the gene level.

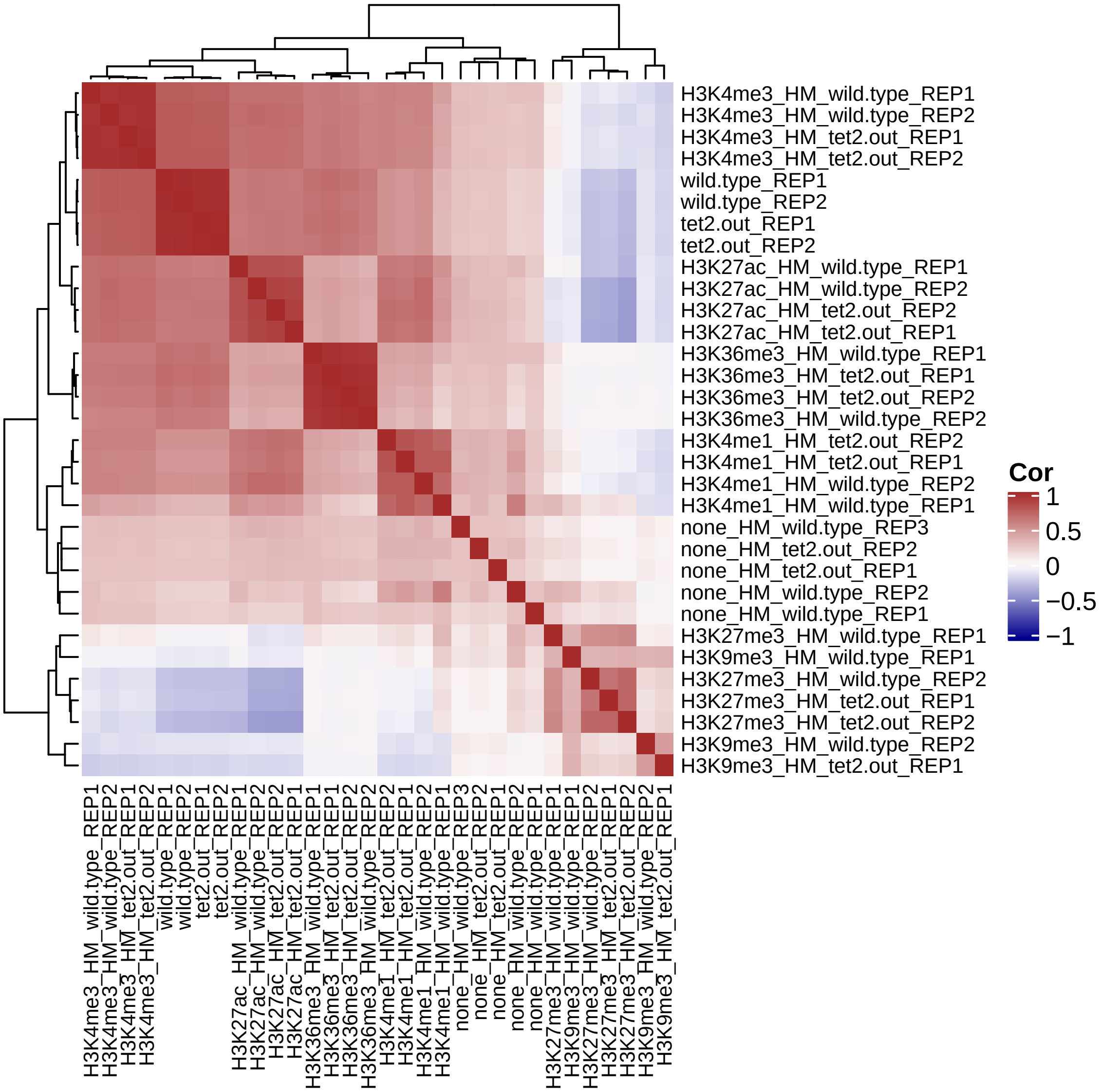

Figure 1: Correlation of RNA-Seq and ChIP-Seq

2. integration: Integrate RNA-Seq and ChIP-Seq data by calculating logFoldChange and Z scores

After the match of RNA-Seq and ChIP-Seq at the gene level, the integration of these two types of data is conducted through the **doIntegration** function by calculating logFoldChange of RNA-Seq and ChIP-Seq and then calculate Z scores for each marker. The input of the function is a list result from **doMatch** function.

```
df_final <- doIntegration(res = res, # result list from "doMatch" function
                          type = "apeglm", # shrinkage estimator, default is "apeglm"
                          ref = "wild.type", # specifying the reference level
                          apeAdapt = FALSE)
```

The result of the second step from **doIntegration** function contains the logFoldChange of RNA-Seq and ChIP-Seq as well as the Z score of each mark for each gene (shown as the table below).

Table 1: integration results

	z.H3K9me3	z.none	z.H3K27me3	z.H3K27ac	z.H3K4me3	z.H3K4me1	z.H3K36me3
0610009B22Rik	1.5096313	-0.4556445	1.1294972	-0.0600427	0.1922781	-0.3990826	0.1116033
0610010F05Rik	0.0727513	-0.3159894	0.1848376	1.2379816	-0.3030021	0.1750952	-0.1077852
0610010K14Rik	0.2818030	0.5665779	0.3684958	0.1205920	0.0840617	0.0560468	0.0950096
0610012G03Rik	-0.0402419	-0.0577281	-0.0344298	0.0053385	0.0248794	0.0119307	-0.0709771
0610030E20Rik	-0.2843424	-0.0069848	-0.0345165	0.5311879	-0.1859840	-0.0157660	-0.1489075
0610040J01Rik	0.0263985	-0.6136573	0.0738724	1.2450397	0.0459432	-0.0293661	0.3395275
1110002E22Rik	-0.1117175	-0.6161363	0.3220724	-0.0374538	0.0682505	-0.4427649	0.4736603
1110004F10Rik	-0.0075126	0.0382641	-0.0531325	-0.0302648	0.0218445	0.0215032	0.0285630
1110008P14Rik	0.5878211	0.1613085	-0.0158789	-0.0216326	-0.1131650	0.1088885	0.2975963
1110012L19Rik	-0.7605611	-0.2186961	-0.4329753	-0.3474599	-0.1035559	-0.2430795	0.3229549
1110017D15Rik	-0.5635032	-0.0215326	0.1116994	-0.2396299	0.2999805	0.4402020	-0.2901453
1110032A03Rik	-0.0308223	1.3990756	-0.3721691	0.5582615	-0.7094221	-0.9638899	0.4072242
1110032F04Rik	-0.0619083	0.2234994	-0.0579622	0.1148733	0.0706187	0.1086061	0.1350982
1110051M20Rik	0.0778253	-0.0865843	-0.2830442	-0.1500254	0.1005083	-0.2821450	0.0665700
1110059E24Rik	-0.0749205	-0.1335756	0.0250346	0.2520036	-0.1724477	-0.2854025	-0.1534097

3. prioritization: prioritization of genes based on Z scores with Pareto optimization

Take the Z scores of several different histone modifications as input, the prioritization of genes based on Z scores can be formulated as multiobjective optimization problem and solved with Pareto optimization [8]. The aim of Pareto optimization method is to find the Pareto optimal trade-off (Pareto front) between conflicting objectives (such as minimizing Z score of H3K27me3 and maximizing z-score of H3K4me3 for each gene). The results of Pareto optimization method is a rank-ordering of the genes by the level of the congruent changes in RNA-Seq and ChIP-Seq (shown as table below).

```
nr.fronts <- 50 # choose a large number to include all the fronts
objective <- data.frame(mark = c("z.H3K27ac", "z.H3K4me3", "z.H3K4me1",
                                 "z.H3K36me3", "z.H3K9me3", "z.H3K27me3"),
                        obj=c("max","max","max","max","min","min"),
                        stringsAsFactors=FALSE)
res_final <- doPareto(df_final = df_final,
                      objective = objective,
                      nr.fronts = nr.fronts)
```

Table 2: prioritization results

	z.H3K9me3	z.none	z.H3K27me3	z.H3K27ac	z.H3K4me3	z.H3K4me1	z.H3K36me3	front
Atp6v0c-ps2	16.589667	3.2706193	-20.013204	-1.725424	11.8417083	-0.5113998	10.440533	1
Catsperg1	-8.061640	-1.5505929	1.034593	10.164148	12.7109471	0.8538197	12.002940	1
Fam110c	7.090283	4.5737313	-3.192932	38.702950	7.4691643	4.8392381	12.702844	1
Grb14	-3.354402	2.4001542	-14.829860	27.547845	10.8787232	4.9987109	15.616601	1
Khdc1a	-9.654657	-5.3475747	3.041933	17.776863	11.8376748	20.2619271	14.673243	1
Metrn	-8.142343	-6.7852712	-5.716880	-10.044830	8.9894024	11.5985664	12.760880	1
Olfr94	-5.706979	0.4598344	-36.221443	99.403578	21.5264205	20.2764413	1.578383	1
Olig1	-6.158681	11.2312845	-20.694139	34.781308	20.0243582	8.6956798	4.906146	1
Pramef17	5.581220	8.4221983	-6.412906	11.671704	6.1742990	21.5723380	21.153664	1
Trav5-2	-8.224873	2.4279795	-3.729377	23.326949	-0.1153585	13.0210337	18.820450	1
Trim34b	-10.368544	-0.9556500	-14.980885	5.767722	3.5631355	-11.3481747	-6.496720	1
Tspo2	-3.858320	-5.9325064	-4.143173	13.234274	30.3344625	12.5968673	38.445580	1
Ttll11	-3.243813	0.0535220	1.956712	98.616938	7.3652294	7.1142097	18.991588	1
Ubd	-26.955101	8.3544039	-12.420790	73.875377	115.7006521	42.1527121	11.752621	1
Zfp786	-6.054174	2.8041174	-0.747937	6.618457	3.1202473	7.2548820	5.319367	2

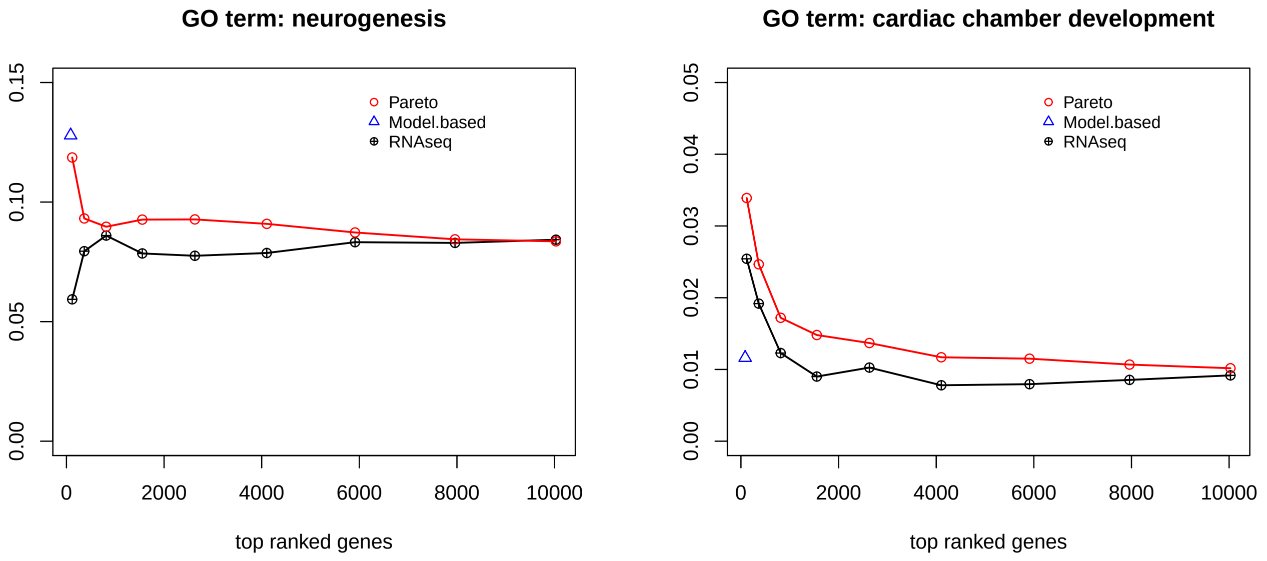

Figure 2: Compare intePareto with another approach and RNAseq alone

Compare our integrative results with other approach and RNA-Seq alone

To evaluate the performance of our method we did the integrative analysis with a recent published the hierarchical Bayesian model-based clustering approach (model-based approach) [9] and analysis of RNA-Seq alone, a functional quality metric, enrichment score of interesting terms for each data set is used to do comparison research between our integrative approach, the model-based approach and the analysis of RNA-Seq data alone. The enrichment score is defined as $N(G_i \cap G_r) \mid N(G_r)$, in which G_i stands for the genes in the interesting GO terms, G_r stands for the genes in the result of analysis.

Both our integrative approach and the model-based approach found that the genes in the final result or the top rank gene (intePareto) enriched in “limb morphogenesis” GO term [Figure 2], which is consist with results in a recently published research [7]. Our approach also found genes in the top-ranked list enriched in “mammary gland formation” which is discussed in this review [3]. This result will be hidden when RNA-Seq data alone was analyzed or the model-based approach was used. When compared to the model-based approach[9], our method outperformed it by having comparable results at the top genes and offering more informative results by providing the rank-ordered list of the remaining genes.

```
sessionInfo()
```

```
## R version 3.6.1 (2019-07-05)
## Platform: x86_64-pc-linux-gnu (64-bit)
## Running under: Ubuntu 16.04.6 LTS
##
## Matrix products: default
## BLAS:   /usr/lib/libblas/libblas.so.3.6.0
## LAPACK: /usr/lib/lapack/liblapack.so.3.6.0
##
## locale:
##  [1] LC_CTYPE=en_US.UTF-8       LC_NUMERIC=C
##  [3] LC_TIME=de_DE.UTF-8        LC_COLLATE=en_US.UTF-8
##  [5] LC_MONETARY=de_DE.UTF-8    LC_MESSAGES=en_US.UTF-8
##  [7] LC_PAPER=de_DE.UTF-8       LC_NAME=C
##  [9] LC_ADDRESS=C               LC_TELEPHONE=C
## [11] LC_MEASUREMENT=de_DE.UTF-8 LC_IDENTIFICATION=C
```

```
##
## attached base packages:
## [1] grid      stats     graphics  grDevices utils     datasets  methods
## [8] base
##
## other attached packages:
## [1] knitr_1.24           circlize_0.4.8       ComplexHeatmap_2.0.0
## [4] intePareto_0.1.1     apeglm_1.6.0
##
## loaded via a namespace (and not attached):
##   [1] bitops_1.0-6                matrixStats_0.55.0
##   [3] bit64_0.9-7                 httr_1.4.1
##   [5] RColorBrewer_1.1-2          progress_1.2.2
##   [7] GenomeInfoDb_1.20.0         numDeriv_2016.8-1.1
##   [9] tools_3.6.1                 backports_1.1.4
##  [11] R6_2.4.0                    rpart_4.1-15
##  [13] Hmisc_4.2-0                 DBI_1.1.0
##  [15] lazyeval_0.2.2              BiocGenerics_0.30.0
##  [17] colorspace_1.4-1            GetoptLong_0.1.7
##  [19] nnet_7.3-12                 prettyunits_1.0.2
##  [21] tidyselect_1.0.0            gridExtra_2.3
##  [23] DESeq2_1.24.0               bit_1.1-14
##  [25] compiler_3.6.1              Biobase_2.44.0
##  [27] htmlTable_1.13.1            DelayedArray_0.10.0
##  [29] scales_1.0.0                checkmate_1.9.4
##  [31] genefilter_1.66.0           stringr_1.4.0
##  [33] digest_0.6.20               Rsamtools_2.0.0
##  [35] foreign_0.8-72              rmarkdown_1.15
##  [37] XVector_0.24.0              base64enc_0.1-3
##  [39] pkgconfig_2.0.2             htmltools_0.3.6
##  [41] highr_0.8                   bbmle_1.0.20
##  [43] GlobalOptions_0.1.0         htmlwidgets_1.3
##  [45] rlang_0.4.5                 rstudioapi_0.10
##  [47] RSQLite_2.1.2               rPref_1.3
##  [49] shape_1.4.4                 BiocParallel_1.18.1
##  [51] acepack_1.4.1               dplyr_0.8.3
##  [53] RCurl_1.95-4.12             magrittr_1.5
##  [55] GenomeInfoDbData_1.2.1      Formula_1.2-3
##  [57] Matrix_1.2-17               Rcpp_1.0.2
##  [59] munsell_0.5.0               S4Vectors_0.22.1
##  [61] stringi_1.4.3               yaml_2.2.0
##  [63] MASS_7.3-51.4               SummarizedExperiment_1.14.1
##  [65] zlibbioc_1.30.0             plyr_1.8.4
##  [67] blob_1.2.0                  parallel_3.6.1
##  [69] crayon_1.3.4                lattice_0.20-38
##  [71] Biostrings_2.52.0           splines_3.6.1
##  [73] annotate_1.62.0             hms_0.5.1
##  [75] locfit_1.5-9.1              pillar_1.4.2
##  [77] igraph_1.2.4.1              GenomicRanges_1.36.0
##  [79] rjson_0.2.20                geneplotter_1.62.0
##  [81] biomaRt_2.40.5              stats4_3.6.1
##  [83] XML_3.98-1.20               glue_1.3.1
##  [85] evaluate_0.14               latticeExtra_0.6-28
##  [87] data.table_1.12.2           RcppParallel_4.4.3
```

```
## [89] png_0.1-7                  vctrs_0.2.4
## [91] gtable_0.3.0               purrr_0.3.2
## [93] clue_0.3-57                assertthat_0.2.1
## [95] ggplot2_3.2.1              emdbook_1.3.11
## [97] xfun_0.9                   xtable_1.8-4
## [99] coda_0.19-3                survival_2.44-1.1
## [101] tibble_2.1.3              GenomicAlignments_1.20.1
## [103] AnnotationDbi_1.46.1      memoise_1.1.0
## [105] IRanges_2.18.3            cluster_2.1.0
```

References

[1] Simon Andrews et al. *FastQC: a quality control tool for high throughput sequence data.* 2010.

[2] Nicolas L Bray et al. "Near-optimal probabilistic RNA-seq quantification". In: *Nature biotechnology* 34.5 (2016), p. 525.

[3] Holly Holliday et al. "Epigenomics of mammary gland development". In: *Breast Cancer Research* 20.1 (2018), pp. 1–11.

[4] Gary C Hon et al. "5mC oxidation by Tet2 modulates enhancer activity and timing of transcriptome reprogramming during differentiation". In: *Molecular cell* 56.2 (2014), pp. 286–297.

[5] Heng Li and Richard Durbin. "Fast and accurate short read alignment with Burrows–Wheeler transform". In: *bioinformatics* 25.14 (2009), pp. 1754–1760.

[6] Heng Li et al. "The sequence alignment/map format and SAMtools". In: *Bioinformatics* 25.16 (2009), pp. 2078–2079.

[7] Rong Li et al. "TET2 Loss Dysregulates the Behavior of Bone Marrow Mesenchymal Stromal Cells and Accelerates Tet2-/–Driven Myeloid Malignancy Progression". In: *Stem cell reports* 10.1 (2018), pp. 166–179.

[8] Patrick Ngatchou, Anahita Zarei, and A El-Sharkawi. "Pareto multi objective optimization". In: *Proceedings of the 13th International Conference on, Intelligent Systems Application to Power Systems.* IEEE. 2005, pp. 84–91.

[9] Martin Schäfer, Hans-Ulrich Klein, and Holger Schwender. "Integrative analysis of multiple genomic variables using a hierarchical Bayesian model". In: *Bioinformatics* 33.20 (2017), pp. 3220–3227.
